# Impact of sustainable project management on project plan and project success of the manufacturing firm: Structural model assessment

**DOI:** 10.1371/journal.pone.0259819

**Published:** 2021-11-24

**Authors:** Tay Chze Chow, Suhaiza Zailani, Muhammad Khalilur Rahman, Zhang Qiannan, Miraj Ahmed Bhuiyan, Ataul Karim Patwary

**Affiliations:** 1 Faculty of Business and Accountancy, University of Malaya, Kuala Lumpur, Malaysia; 2 Faculty of Entrepreneurship and Business, Universiti Malaysia Kelantan, Pengkalan Chepa, Malaysia; 3 School of Economics, Guangdong University of Finance and Economics, Guangzhou, China; 4 Faculty of Hospitality, Tourism and Wellness, Universiti Malaysia Kelantan, Pengkalan Chepa, Malaysia; Universiti Pertahanan Nasional Malaysia, MALAYSIA

## Abstract

This study has aimed to investigate the impact of sustainable project management on sustainable project planning and success in manufacturing firms. Data was collected from project management professionals in a manufacturing firm in Malaysia. A total of 231 responses were analyzed using the partial least square (PLS) method. The findings revealed that sustainable project management has a significant impact on sustainable project success and sustainable project planning. Sustainable project planning is positively correlated with sustainable project success. The results also indicated that sustainable project planning mediates the effect of sustainable project management on sustainable project success. The findings have significant insight into the body of knowledge of the project life cycle and indicated that sustainable project planning is a crucial tool attributed to project management towards the project success of the manufacturing firm. The results can be used as a guideline for organizations, providing direction in project management to achieve sustainable development for business.

## Introduction

Sustainability is the capacity to be maintained at a certain level. In this study, sustainability refers to project management’s economic, environmental, and social benefits in a manufacturing firm. Sustainability is an integral part of project management practices that maintain the economic, environmental, and social (triple bottom line) future benefits. Kivilä et al. [1] indicated sustainable project management (SPM) with Triple Bottom Line (TBL)—economic, environmental, and social has a significant impact on project success. SPM focuses on the planning, monitoring, controlling, and ensuring project delivery process along the project life cycle. Project managers are responsible for looking at the overview of project management with the integration of sustainability as one of the project’s objectives [2–4]. Sustainable project management can lead to maintaining sustainable project planning, which reflects the manufacturing firm’s sustainable project success. The integration of sustainability into project planning practices is essential to ensure the project management process and planning. In this study, sustainable project planning (SPP) comprises three main dimensions: managerial control, risk response, and work consensus.

In Malaysia, the manufacturing firm lacks innovation, competitiveness, labor-intensive industries, and inadequate enablers [5]. Tay et al. [6] stated that due to limited raw material, data storage capacity, handling variability, and streaming stability, manufacturing companies in Malaysia lack comprehensive business sustainability. Business sustainability is often defined as managing the triple bottom line. Terrafiniti [7] reported that the work of sustainability managers is slowly entering established practice in the manufacturing industry. Many organizations have adopted a sustainability approach in Malaysia; however, there is still great variability in practice, and sustainability managers and project planners are often hampered by resistance, apathy, and misunderstanding.

The role of project planning facilitates project management throughout the project life cycle and leads to the project’s success [8]. To encourage the integration of sustainability [9] in project management and project planning, sustainability measurement dimensions need to be added as a project success criterion. There are six dimensions to evaluate sustainable project success (SPS), project efficiency, stakeholders, team, business success, preparation for the future, and sustainability. The main objective of this study is to explore what are the sustainable measurement dimensions related to sustainable project management to predict sustainable project planning and sustainable project success in the manufacturing industry.

Alsawafi et al. [10] and Elkington [11] explained the concept of sustainability business, which is known as the Triple Bottom Line (TBL): Economic, Environmental, and Social. TBL is a measurement of performance incorporate. TBL can lead manufacturing companies to focus on social and environmental concerns and generate profits [12]. The manufacturing companies face pressure in operating their business activities [13, 14]. This is because of the unstable of economic (recession), environmental (depletion of natural resources), and social (labors and human rights) issues [11]. Sustainable project management can change the policies of the business organizations to achieve specific objectives as success criteria [15]. The governments highly acknowledge the responsibility to adopt sustainability into project development strategies in many countries due to increases in population and limited resources, especially in the area of sustainable development [16]. Consequently, a sustainable project management mechanism can lead to maintaining a sustainable project plan, leading to sustainable project success for the manufacturing companies. This study emphasized that sustainable project management is a crucial tool that predicts sustainable project plans and sustainable project success.

## Literature review

### Underpinning theory

This study has adopted the concept of sustainability in project management [16]; developed the relationship between sustainable project management and project success. Yu et al. [17] stated that sustainable project success is a key determinant that leads to the success of projects of the companies. Following the concept of sustainable project management [16], we propose an approach that considers sustainability from the triple bottom line (TBL) perspective (e.g., economic, environmental, and social). In this study, sustainable project management (economic, social, and environment) reflects sustainable project planning (managerial control, risk response, work consensus), and sustainable project success (efficiency, team business success, preparation for future, sustainability). Shokri-Ghasabeh and Kavoousi-Chabok [18] indicated that sustainable project management contributes toward the success of project encourage more organizations to practice sustainability in project management, as sustainability is one of the measurements in project success.

### Sustainable project management

Project management is the processes, methods, knowledge, skills, and experience to achieve specific project objectives. Sustainable project management refers to implementing projects that will serve to support future generations and society in economic, environmental, and social benefits [19]. This study evaluates sustainable project management by the manufacturing firm’s economic, environmental, social benefits. Reducing the use of natural resources, liquid waste, biodiversity, and energy can lead to sustainable project management. Besides, the manufacturing firm or company’s relationship with the local community, labor practices management, and human rights management is a crucial element that assists in maintaining sustainable project management. Sustainable development has been widely promoted and has started a new development paradigm in governmental and non-governmental organizations. Many organizations are moving towards sustainability in project management, which could incorporate sustainable project planning and sustainable project success of the company.

Sustainability has also moved towards changing the profession in project management. The researchers explored the importance and practices of sustainability in project management [20–22]. Martens and Carvalho [23] suggested that the sustainability principle with TBL dimensions (economic, environmental and social) should be included in the project management process, which leads to integrating sustainable project planning and contributing to business success organizations. Martens and Carvalho [24] explained the challenges of sustainability in the project management function. Dvir et al. [25] dispute that project planning and project success should also be included in the sustainable project management context. Martens and Carvalho [23] analyzed the sustainable project management from different manufacturing industries and applications and clustered it into the TBL (e.g. economic, environmental and social) dimensions. In their study, the main concerns of the economic variable are the company’s financial performance and its advantages from social and environmental practices, cost management, stakeholder management, and business ethics in economic performance. Thus, we postulated that:

H1: Sustainable project management has a significant impact on sustainable project success.
H2: Sustainable project management has a significant impact on sustainable project planning.

### Sustainable project planning and success

Project planning contains the project activities [25], schedule, cost and resources planned within the project life cycle of the business organization. This study emphasizes sustainable project planning, which is evaluated by managerial control, risk response, and work consensus in the manufacturing firm. The managerial control, project task, process, and solution for potential risks are the crucial elements for measuring sustainable project planning. Sustainable project success refers to the development that meets the needs of the present. This study considers project efficiency, stakeholders, team, business success, preparation for the future, and sustainability to evaluate the manufacturing firm’s sustainable project success. For the sustainability of project success, economic costs and benefits of government policy or business strategy are required to be taken into consideration. The key elements of project efficiency (e.g. cost or budget, completion on time, and scope of the project) consist of sustainable project success. The technical specification of project meetings, solving customers’ problems, and improving customers’ quality of life are crucial components for evaluating sustainable project success. Besides, the realization and perpetuation of economic, environmental, and social benefits are major elements for the success of the sustainable project. The team’s productivity, profitability, market share, and new technologies can lead to sustainable project success for the manufacturing firm.

In this study, sustainable project success refers to the company’s efficiency, stakeholder, teamwork, and preparation for future business success. Project planning is the most critical step to be performed in the project management process [26] and sustainable project success in the company. Managing a project is challenging, as it is challenging to construct a project plan suitable for all types of the project due to different projects operation. Therefore, project monitoring and controlling should be applied to adapt to the fast-changing situation in a project environment. Some researchers argue that sustainability in project planning can help moderate the dynamic project environment by reducing the uncertainties and pre-determine underlying problems within the project context [27]. Project risk is reviewed during the planning process, and risk management help to mitigate the high-risk activities; thus, the project uncertainties can be a remedy through the planning process [28]. Besides, proper detailed planning allows a project team to understand the project objectives clearly, and lead the behavior of a project to improve the efficiency of the execution. The previous literature explored sustainable project planning efforts that affect project success [17]. However, an integration of sustainability in project planning into sustainable project management and the impact on sustainable project success has yet to be realized and put into a research model. Therefore, we postulated that:

H3: Sustainable project planning has a significant impact on sustainable project success.

### Mediating effect of sustainable project planning

The project success corresponds to good project management, which comprises the objectives and benefits envisioned by the project team. It is good to include sustainability in the initial objectives, thus enabling the organization to enjoy the sustainable project outcome. For a project-oriented company, Kerzner [29] stated that project success is closely related to the results through the projects, as these are the company’s fundamental business and core competencies. The performance measurement and planning ability are also considered part of the measurements for project management, which contribute to the success of projects [30]. Project Management Institute [15] explained the measures of project management time, cost, scope and quality, resources, and risk, which are widely applied in project management for the company’s success. The previous studies [16, 23, 24, 31] extended and classified the dimensions of project success such as project efficiency, impact on the customer, team, business success, preparation for the future, and sustainability. Dvir et al. [25] examined the relationship between sustainable project management and sustainable project success. Carvalho et al. [32] stated that the sustainability dimensions in economic, environmental, and social contexts could influence the manufacturing company’s project success. Thus, we postulated that:

H4: Sustainable project planning mediates the effect of sustainable project management on sustainable project success.

**Fig 1:**
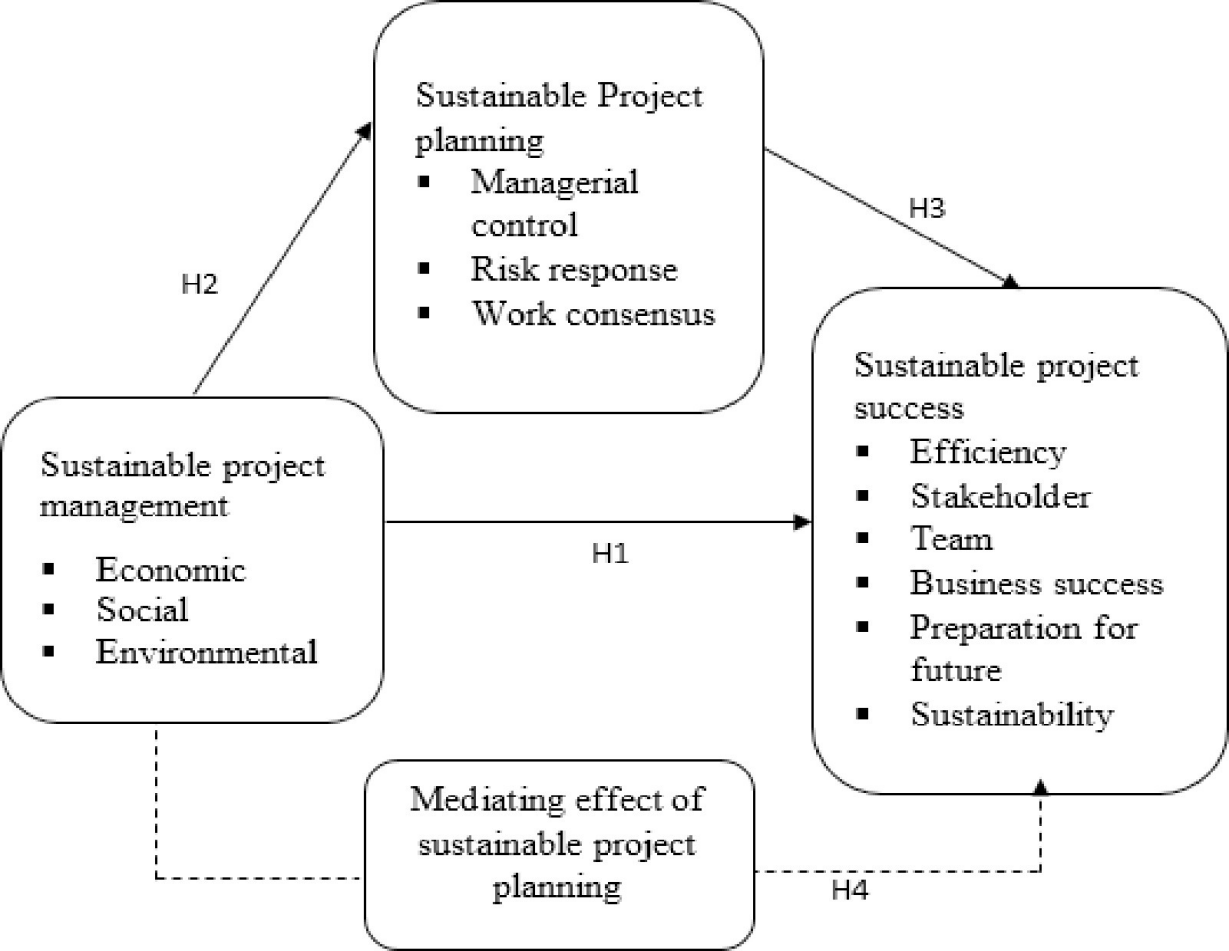
Conceptual model.

Based on the review of literature and concept of underpinning theory, Fig 1 shows the conceptual model of this study.

## Methodology

### Operationalization of construct

The measurement instruments of this study were adopted and modified from the review of the literature. In this study, sustainable project management consists of three major dimensions of the triple bottom line (TBL) such as economic, social and environmental, which measured with nine items adopted from Martens and Carvalho [23], using a five-point Likert scale ranging from 1 (unimportant) to 5 (very important). Similarly, sustainable project planning is entailed with three dimensions such as managerial control, risk response, and work consensus, which evaluated with ten items modified from [17] and used a five-point Likert scale ranging from 1–5, whereas 1 for "very little extent", 2 for "little extent", 3 for "some extent", 4 for "great extent" and 5 for "very great extent". Sustainability project success is contained five dimensions such as efficiency, stakeholder, team, business success, preparation for future, and sustainability in this study, which measured with eighteen items modified from Martens and Carvalho [24], using the five-point Likert scale ranging from 1 (very little extent) to 5 (great extent).

### Data collection and sampling method

We used a self-administered questionnaire for distributing to the respondents using an online survey form. For distributing questionnaires, the online survey method is easy to reach the respondents through the internet [33]. The respondents’ addresses are obtained from the Project Management Institute (PMI). PMI is a well-known global non-profit project management organization for project management. In this study, the respondents’ list is limited to members of the PMI in Malaysia. Based on PMI Malaysia’s official website, 1320 people were registered as members of the PMI Malaysia Chapter (*PMIMY*) in 2019 [34]. We used a stratified random sampling method for collecting data. We collected the profile and email ID of the project manager, engineer, director, and CEO of the manufacturing company from the site of *PMIMY*. We distributed the questionnaire with a consent form and politely request the respondents to participate in the study. We ensured the respondents that the survey has been conducted only for academic purposes, and there is no personal identification, and responses will remain anonymous.

We distributed the questionnaires with Sekaran and Bougie [33] suggested that it is suitable for selecting the participants in this study due to different levels of management having another point of view in project management. Data was collected from the low-level project management (e.g., Project Engineer), middle-level management (e.g., Project Managers and Senior Project Managers), and top management (e.g., Managing Director and CEO) of the manufacturing firm in Malaysia. Online survey questionnaire links were distributed through social media platforms such as LinkedIn and WhatsApp group. A total of 300 questionnaires with survey links were distributed to respondents. We received a total of 238 return questionnaires out of 300. During the data screening process, we identified 7 incomplete responses, and thus, 231 valid responses were received for data analysis with a response rate of 77%. To evaluate the reliability of the sample size, we used G-Power 3.1 statistical tool. The results indicated with the effect size of 0.15, error 0.05, and the number of predictor 2, G-Power suggest sample size of 107 with the actual power of 0.95 to examine the conceptual model. Reinartz et al. [35] postulated that using the partial least square (PLS) method, the minimum sample size is required 100. In this study, we have collected 231 valid responses from the manufacturing firm, exceeding the minimum sample size requirement. Thus, the sample size of this study is acceptable and adequate for the analysis.

### Common method variance

Common method variance plays a significant role in social science studies due to the single source of data. Podsakoff et al. [36] postulated that Harman’s single-factor test is crucial to measure the common method variance. In this study, we used Harman’s [37] single-factor test. The result indicated that the highest factor estimated 21.53% of the variance, which is lower than 50%, indicating no common method variance in this study.

## Findings

### Demographic information

The demographic information and outcome of the responded are shown in Table 1. The results revealed that Middle-level management ranked the highest at 57.2% of the total sample size. Meanwhile, 36.8% of respondents are holding low-level management. The rest 6.0% are holding a significant position (high-level management). In academic qualification, the majority of the respondents are bachelor’s degrees (67.3%) followed by 31.7% respondents with master’s degrees, and only 1% respondent is Doctoral degrees. Most of the respondents have less than 5 years in project management experiences of 35.6%; while 32.7% of respondents have 6 to 10 years of project management experience and 11.9% and 10.9% of respondents had have 11 to 15 years and 16–20 years of project management experiences, and rest 8.9% of respondents have over 20 years of project management experiences. In the highest project *Capital expenditures* (CAPEX) category, 47.5% of respondents have experience in managing over RM20 million of project value in a single project. Following by the second "less than RM5 millions" and third "less than RM1 mil" rank of the CAPEX amount with 39.6% of respondents, followed by 8.9% not exceeding RM10 million and 4.0% of them is not exceeding RM20 million of highest project capital expenditure.

**Table 1:** Profile of respondents.

Variables	Description	Percentage (%)
Designation	Low-Level Management (Executives)	36.8%
	Middle Level Management	57.2%
	Top Level Management (Owner, CEO, MD, GM, Director)	6.0%
Academic Qualification	Secondary School	0.0%
	Diploma/STPM/A-level or Equivalent	0.0%
	Bachelor’s degree	67.3%
	Master’s degree	31.7%
	Doctoral Degree/ PhD	1.0%
Project Management Experiences	Less Than 5 Years	35.6%
	6–10 Years	32.7%
	11–15 Years	11.9%
	16–20 Years	10.9%
	More Than 20 Years	8.9%
Highest Project Capital Expenditure (CAPEX)	Not Exceeding RM 1,000,000	17.8%
	Not Exceeding RM 5,000,000	21.8%
	Not Exceeding RM 10,000,000	8.9%
	Not Exceeding RM 20,000,000	4.0%
	More Than RM 20,000,000	47.5%

Table 2 illustrates the information of respondents’ firm, which consists of the industry category of the company, numbers of employees in a firm, ownership status, company’s annual sales turnover amount, and practicably of sustainability in business or project management. The distribution of industry category is mainly on petrochemical industry 20.8% of respondents, following by construction 19.8% and 18.8% for information technology (IT) and telecommunication industry. About 13.9% of respondents are working in the consultancy industry, while 7.9% of respondents are in the chemical/gas industry. This is followed by accounting/finance, health care, and manufacturing industries, which sharing of 9.0% equally. The remaining industry contributed 9.8%, which are logistics, machine makers. Regarding respondents’ firm, the number of employees was 35.6%, more than 1000 employees, which is considered a larger company in Malaysia. This is followed by 26.7% less than 100, 12.9% from 101–250, and 501–1000. The least proportion of 11.9% of respondents works in a firm that consists of 251–500 employees. In terms of firm ownership, approximately 59.4% of respondents work in private-owned companies, 37.6% work in publicly listed companies, and 3.0% work in government-linked companies and non-governmental organizations. Most of the company’s annual sales turnover amount over RM100 million (46.5%), 26.7% of the company have RM500k to RM20 million annual sales turnover, 19.8% has RM50 million to RM100 million, and 4.8% of respondent’s firm has RM20 million to RM50 millions yearly sales turnover, and 2.0% of them has less than RM500k. For a firm’s sustainability practice, 75.2% of the company practice sustainability in business or project management, while the remaining 24.8% companies are not.

**Table 2:** Profile of respondents’ firm.

Variables	Description	Percentage (%)
Industry of Company	Accounting/Finance	3.0%
	Chemical/Gas	7.9%
	Construction	19.8%
	Consultancy	13.9%
	Health Care	3.0%
	Information Technology (IT)/	18.8%
	Manufacturing	3.0%
	Petrochemical	20.8%
	Others (Logistics, Machine, etc.)	9.8%
Number of Employees	Less Than 100	26.7%
	101–250	12.9%
	251–500	11.9%
	501–1000	12.9%
	More Than 1000	35.6%
Ownership Status	Private Owned Company	59.4%
	Public Listed Company	37.6%
	Government Linked Company	3.0%
	Non-Governmental Organization	0.0%
Company’s Annual Sales Amount	Less Than RM 500,000	2.0%
	RM 500,001—RM 20,000,000	26.7%
	RM 20,000,001—RM 50,000,000	5.0%
	RM 50,000,001—RM 100,000,000	19.8%
	More Than RM 100,000,001	46.5%
Company Practice on SPM	Yes	75.2%
	No	24.8%

### Measurement model assessment

SmartPLS 3.0 software is used through the structural equation modelling approach to assess the model of this study, and reliability analysis. The findings revealed that composite reliability (CR) ranged from 0.831–0.953, while the accepted threshold value is >0.70 [38]. It implies that all items are sufficient to represent the respective constructs and all constructs are reliable. The Rho_A is reflected in the range between 0.758 and 0.945, the threshold value is >0.70 [39], which satisfy the internal consistency reliability. The factor loading ranged (FL) from 0.725–0.939, which is greater than 0.60 [38], thus, it disclosed sufficient internal reliability. The average variance extracted (AVE) for all variables shown in ranging from 0.498 to 0.869 (Table 3). Hence, the AVE value for the economic dimension shows 0.498 that is close to the cut-off point of 0.50 [38]. In addition, other criteria such as factor loading, CR, and Rho_A values are achieved the satisfactory level except for the AVE value. Thus, it suggests that the study explained adequate convergent validity [39, 40]. The findings of variance inflation factor (VIF) are ranged between 1.384 and 2.980, which is less than cut-off point 5. Hair et al. [40] believed that collinearity issues may occur if VIF values exceed 5. Thus, the findings indicating that the multicollinearity issue does not exist in this study. The measurement items are included in Appendix.

**Table 3:** Convergent validity.

1^st^ Order	2^nd^ Order	Items	FL	CR	AVE	Rho_A
Sustainable Project Management (SPM)	Economic (TECON)	Econ01	0.832	0.831	0.498	0.758
		Econ02	0.792			
		Econ03	0.738			
	Social (TSOC)	Soc01	0.725	0.927	0.682	0.908
		Soc02	0.879			
		Soc03	0.923			
	Environmental (TENV)	Env01	0.825	0.927	0.719	0.945
		Env02	0.827			
		Env03	0.883			
Sustainable project planning (SPP)	Managerial control (TMC)	MC01	0.784	0.872	0.579	0.820
		MC02	0.852			
		MC03	0.810			
	Risk Response (TRR)	RR01	0.776	0.899	0.640	0.862
		RR02	0.836			
		RR03	0.816			
	Work Consensus (TWC)	WC01	0.845	0.896	0.682	0.845
		WC02	0.856			
		WC03	0.808			
		WC04	0.793			
Sustainable project success (SPS)	Project Efficiency (TPE)	PE01	0.854	0.886	0.721	0.807
		PE02	0.882			
		PE03	0.811			
	Stakeholder (TISE)	ISE01	0.915	0.935	0.827	0.895
		ISE02	0.922			
		ISE03	0.891			
	Impact on team (IMT)	IMT01	0.824	0.907	0.765	0.858
		IMT02	0.912			
		IMT03	0.885			
	Business success (TBS)	BS01	0.925	0.952	0.869	0.925
		BS02	0.939			
		BS03	0.933			
	Preparation for future (TPPF)	PPF01	0.909	0.922	0.797	0.873
		PPF02	0.876			
		PPF03	0.893			
	Sustainability (TSUS)	SUS01	0.864	0.921	0.796	0.875
		SUS02	0.892			
		SUS03	0.920			

**Note: FL (**Factor loading), CR (Cronbach’s alpha), AVE (Average variance extracted).

The results of the Fornell and Larcker [41] criterion for this study is shown in Table 4. The square root of the AVE each construct (diagonal) exceeded and it is highest among the other inter-correlation constructs (horizontal axis), which explained that all constructs share more variance with their associated indicators than with any other construct [38], which indicated a positive result in supporting discriminant validity.

**Table 4:** Discriminant validity.

	1	2	3	4	5	6	7	8	9	10	11	12
1.TECON	0.712											
2.TENV	0.512	0.848										
3.TSOC	0.620	0.468	0.826									
4.TMC	0.431	0.328	0.325	0.761								
5.TRR	0.334	0.190	0.453	0.544	0.800							
6.TWC	0.234	0.321	0.325	0.593	0.588	0.826						
7.TPE	0.172	0.120	0.193	0.341	0.464	0.464	0.849					
8.TISE	0.279	0.293	0.256	0.343	0.402	0.484	0.603	0.909				
9.TIMT	0.284	0.300	0.378	0.369	0.386	0.479	0.520	0.501	0.874			
10.TBS	0.187	0.142	0.249	0.359	0.479	0.444	0.683	0.544	0.539	0.932		
11.TPPF	0.286	0.266	0.234	0.370	0.301	0.403	0.492	0.437	0.558	0.667	0.893	
12.TSUS	0.328	0.359	0.423	0.435	0.514	0.568	0.519	0.456	0.589	0.658	0.692	0.892

In the cross-loading criterion, the item loadings are generated using the PLS algorithm function and illustrated in Table 5. The result of all the items cross-loading of the respective construct is higher against the items’ loading of other constructs. The highest item loading for each construct is italic to represent the respective intended constructs. Hence, we can conclude that each construct is more closely correlated to its items than other constructs’ items, signifying the discriminant validity.

**Table 5:** Cross loading.

	TECON	TENV	TSOC	TMC	TRR	TWC	TPE	TISE	TIMT	TBS	TPPF	TSUS
ECON1	0.632	0.310	0.444	0.217	0.232	0.087	0.094	0.172	0.182	0.042	0.107	0.171
ECON2	0.664	0.210	0.340	0.200	0.141	0.115	0.205	0.308	0.242	0.187	0.233	0.126
ECON3	0.792	0.347	0.595	0.430	0.455	0.249	0.293	0.259	0.249	0.237	0.267	0.326
ENV1	0.375	0.825	0.356	0.222	0.194	0.234	0.057	0.230	0.224	0.095	0.172	0.304
ENV2	0.323	0.827	0.362	0.242	0.063	0.176	0.093	0.238	0.310	0.125	0.265	0.299
ENV3	0.401	0.882	0.407	0.279	0.129	0.301	0.109	0.314	0.330	0.135	0.211	0.318
SOC1	0.445	0.448	0.725	0.260	0.342	0.342	0.108	0.147	0.220	0.118	0.136	0.351
SOC2	0.579	0.390	0.879	0.314	0.403	0.254	0.207	0.165	0.448	0.233	0.230	0.364
SOC3	0.598	0.370	0.923	0.313	0.330	0.231	0.118	0.195	0.328	0.174	0.224	0.374
MC1	0.384	0.326	0.264	0.784	0.429	0.480	0.202	0.293	0.301	0.265	0.218	0.263
MC2	0.335	0.243	0.172	0.852	0.421	0.436	0.169	0.217	0.170	0.187	0.213	0.280
MC3	0.344	0.223	0.280	0.786	0.355	0.427	0.165	0.195	0.409	0.291	0.360	0.425
RR1	0.291	0.224	0.330	0.453	0.776	0.409	0.392	0.290	0.281	0.402	0.230	0.409
RR2	0.257	0.250	0.323	0.514	0.835	0.524	0.365	0.350	0.286	0.368	0.308	0.485
RR3	0.326	0.120	0.353	0.446	0.816	0.418	0.287	0.236	0.330	0.339	0.254	0.430
WC1	0.074	0.248	0.161	0.532	0.460	0.845	0.336	0.319	0.341	0.357	0.330	0.412
WC2	0.173	0.263	0.265	0.436	0.417	0.856	0.348	0.444	0.449	0.399	0.348	0.494
WC3	0.184	0.287	0.305	0.376	0.530	0.808	0.389	0.327	0.414	0.361	0.226	0.469
WC4	0.325	0.262	0.337	0.593	0.528	0.793	0.453	0.501	0.381	0.352	0.420	0.498
PE1	0.173	0.125	0.059	0.264	0.388	0.365	0.854	0.538	0.389	0.613	0.477	0.492
PE2	0.054	0.049	0.108	0.226	0.364	0.391	0.882	0.411	0.449	0.587	0.409	0.390
PE3	0.203	0.129	0.331	0.360	0.430	0.426	0.811	0.585	0.490	0.538	0.364	0.438
ISE1	0.198	0.264	0.211	0.298	0.344	0.473	0.569	0.915	0.378	0.496	0.378	0.460
ISE2	0.203	0.204	0.187	0.304	0.343	0.416	0.562	0.922	0.468	0.504	0.373	0.373
ISE3	0.342	0.328	0.300	0.321	0.408	0.430	0.517	0.891	0.519	0.485	0.440	0.413
IMT1	0.353	0.218	0.365	0.325	0.283	0.389	0.402	0.338	0.824	0.327	0.431	0.497
IMT2	0.228	0.327	0.326	0.323	0.352	0.475	0.433	0.445	0.912	0.505	0.469	0.537
IMT3	0.173	0.239	0.310	0.320	0.370	0.392	0.520	0.515	0.885	0.559	0.554	0.513
BS1	0.173	0.139	0.247	0.328	0.439	0.452	0.660	0.492	0.474	0.925	0.629	0.639
BS2	0.149	0.401	0.194	0.359	0.402	0.418	0.644	0.538	0.530	0.939	0.623	0.611
BS3	0.200	0.121	0.259	0.306	0.460	0.372	0.606	0.493	0.504	0.933	0.615	0.591
PPF1	0.170	0.239	0.132	0.333	0.242	0.363	0.494	0.427	0.416	0.619	0.909	0.590
PPF2	0.352	0.155	0.270	0.386	0.365	0.371	0.447	0.384	0.518	0.633	0.876	0.635
PPF3	0.258	0.322	0.225	0.266	0.197	0.346	0.375	0.359	0.563	0.533	0.893	0.629
SUS1	0.292	0.284	0.370	0.427	0.508	0.450	0.526	0.527	0.532	0.650	0.679	0.864
SUS2	0.254	0.409	0.307	0.347	0.405	0.560	0.418	0.324	0.494	0.547	0.581	0.892
SUS3	0.346	0.274	0.452	0.378	0.454	0.515	0.438	0.353	0.547	0.556	0.583	0.920

### Structural model assessment

The hypothesis of the research was tested using the PLS algorithm and bootstrapping approach. The larger R^2^ value implies more accurate and precise constructs. The findings revealed that sustainable project planning explains 21.2% variance, and sustainable project success explains 40.1% variance. The f^2^ values showed 0.269, 0.400, and 0.028 for sustainable project management (SPM), sustainable project planning (SPP) and sustainable project success (SPS) respectively. The results found that SPM has a positive correlation to SPS (β = 0.147, t = 1.938 and p<0.05), therefore, H1 is supported. The relationship between SPM and SPP is found significant (β = 0.460, t = 5.178 and p<0.01), and thus H2 is supported. Furthermore, SPP has the strongest relationship with SPS (β = 0.552, t = 7.321 and p<0.01), and therefore H3 is supported (Table 6). Henseler et al. [42] stated that assessing the direct and indirect relationships between exogenous and endogenous latent variables is important for the evaluation of a structural model. The findings revealed that sustainable project planning mediates the effect of sustainable project management on sustainable project success (β = 0.254, t = 7.321 and p<0.01).

**Table 6:** Path coefficient.

Hypothesis		Beta	SD	t-value	Comment	f^2^	R^2^
H1	SPM → SPS	0.147	0.080	1.938*	Supported	0.028	
H2	SPM → SPP	0.460	0.090	5.178**	Supported	0.269	0.212
H3	SPP → SPS	0.552	0.074	7.321**	Supported	0.400	0.401
Mediating effect							
H4	SPM→SPP→ SPS	0.254	0.060	4.216**	Supported		

Note

*p<0.05 (1.645)

**p<0.01 (above 2.33).

## Discussion

The findings revealed that sustainable project management has a highly significant impact on sustainable project planning. This finding is relevant to [2, 17], highlighted in integrating project management and project planning for a construction engineering project. There is a lack of empirical studies that measure the relationship between sustainable project management and sustainable project planning in Malaysian manufacturing firms. Eid [43] addressed the impact of sustainable development on project management processes, specifically focuses on project management processes from different perspectives (e.g., initiation, planning, execution, controlling, and closure). Yu et al. [17] suggested that sustainability in project planning helps to realize the goal of sustainable project management, specifically in the project life cycle process. However, this study concluded new findings that there is a significant positive correlation between sustainable project management and sustainable project planning. The project planning assists in guiding the project team in execution, controlling and monitoring the project. Sustainable project planning can lead to identifying and minimizing the project risk and communicating with the team and stakeholders who have certain credits contribute to sustainable project management.

The results revealed that there is a significant positive link between sustainable project management and project success. This finding is relevant to [23, 32], who identified a significant positive relationship between sustainability in project management and process success in a different context. Mir and Pinnington [44] indicated that the project management process was implemented with a project life cycle model (planning, execution, monitoring, controlling, and closure) and appropriate planning procedures. Besides, the sustainable project success was evaluated with the project efficiency, impact on stakeholders, team, business success, preparation for future, and sustainability measurement to reflect the integration of sustainability in project management.

Sustainable project planning has a highly significant impact on sustainable project success. It implies that sustainable project planning is an essential tool that affects the manufacturing company’s project success. This finding is relevant to the previous studies [17, 45, 46], whereas project planning was found a significant impact on project success. Zwikael et al. [46] identified project risk as a significant factor that measuring the project efficiency and effectiveness with the presence of risks. It implies that the potential risks, project planning delivery, and solutions for potential risks during the project planning process can reflect sustainable project planning. The adoption of managerial control, risk response, and work consensus can improve the manufacturing firm’s sustainable project planning.

The findings revealed that sustainable project planning mediates the effect of sustainable project management on sustainable project success. It implies that sustainable project planning is a critical factor contributing to sustainable project success in the life cycle of the project management process. In the context of sustainable project management, practising good project planning could lead to sustainable project success for the industry. There is some research on project planning in a project management context, which in turn leads to project success [47]. This study found that sustainable project management is highly correlated to sustainable project planning, which directly reflects success. Moreover, sustainable project planning is highly associated with sustainable project success. It denotes that both sustainable project management and sustainable project planning lead to deliver better sustainable project success for the manufacturing industry.

The findings of this study concluded that sustainable project management could be composed of three essential constructs, with economic, social, and environmental dimensions; these findings are related to previous studies [16, 23] in which the researchers highlighted the key factors of sustainable project management and challenges of sustainable project management functions. Over past decades, researchers conducted an exploratory study on sustainability in project management, project success, and sustainability in project planning separately. Sustainable project management is closely related to project success and integration of project planning[17, 24], and project success. Sustainable project management and planning can lead to sustainable project success for the manufacturing firm. The findings identified that the economic, social and environmental dimensions are crucial for sustainable project management because, manufacturing company’s financial and economic performance, financial benefits, cost management, natural resources, energy, labor practices management, and relationships with the local community can assist to improve the sustainable project management.

## Conclusion

The findings of the study have a crucial implication. Practically, the importance of sustainable project management is essential in improving the success of a project of business organizations. The social, environmental, and economic dimensions can play a significant role in leading the company’s sustainable project management. The relation between sustainable project management and sustainable project planning was evaluated as a new finding. Sustainable project management, including economic, environmental and social planning needs effort on managerial control, risk response, and work consensus during project planning of the business industry. Sustainable project planning can lead to predicting the project success of the industries. The significant relationship between sustainable project planning, sustainability in project management, and project success was identified as a new empirical finding of this study. The findings of this study could contribute to the business organizations in providing direction in project management to achieve sustainable development and success for business organizations. The project managers can evaluate and improve the relationship between sustainable project management (with economic, environmental, and social context) and sustainable project planning for evaluating the success (project efficiency, impact on stakeholders, external impact on team, business success, and preparation for future) of the project. There is required more attention to the role of sustainable project planning on directing and controlling the project management, reducing project risks, and forming the understanding and commitment for the sustainable success of the project.

This study was conducted only based on the project management professional (PMP) and project executives who are working in Malaysia. It implies the limitation of sample size and generalization of the study due to the participation of limited numbers of PMP and project executives in this study. The respondents were not defined based on projects’ experiences, as different project experiences causing different knowledge backgrounds, which may have affected the results. Thus, more explanatory studies can be conducted in the future with different cultures and countries and enhance the generalization of sustainable project management, sustainable project planning, and sustainable project success scale. Future research can focus on the relationship between the importance of sustainable project management, the extent of efforts in sustainable project planning, and the impact on sustainable project success in specific project contexts such as information technology and business-related projects. Future research can be conducted by including other project management professionals (e.g. managers and directors) with large sample sizes or respondents to better generalise the study.

The importance of sustainability in project management is found to be significant in this study. To achieve business objectives, it is crucial to include sustainability in project management with the triple bottom line such as economic, environmental and social. Based on the study’s findings, we identified that there is a highly significant relationship between sustainable project planning and sustainable project success. It implies that sustainable project planning predicts the sustainable project success of manufacturing firms in Malaysia. In addition, the results also indicated that sustainable project management is highly correlated with sustainable project planning. It denotes that sustainable project management is crucial for the success of a sustainable project in the company.

Moreover, there is a positive and significant relationship between sustainable project management and sustainable project success. These findings imply that sustainable project management and planning are the key functions for the development of sustainable project success. Sustainable project planning serves as a bridge to link sustainable project management and sustainable project success. This study emphasizes that sustainable project planning manifested three dimensions: managerial control, risk response and work consensus. Sustainable project planning in the project life cycle can predict project success. It denotes that sustainable project planning is a critical tool to maintain sustainability in project management. The study identified the importance of sustainable project planning in the project life cycle from a sustainable project management perspective, which in turn leads to the manufacturing firm’s sustainable project success. The measurement scale of this study may help the business organization develop sustainability in project management towards sustainable project success through the proper implementation of sustainable project planning.

## Supporting information

S1 Dataset (XLSX)

S2 Dataset (XLSX)

S1 Appendix (DOCX)
