# Supplementary material for: Impact of sustainable project management on project plan and project success of the manufacturing firm: Structural model assessment
Source: PLoS One. 2021 Nov 24;16(11):e0259819. doi: 10.1371/journal.pone.0259819 (PMC8612515; doi:10.1371/journal.pone.0259819)
Supplement: S1 Appendix — (DOCX) [file pone.0259819.s003.docx]

Appendix

| **Constructs** | **Items** | **VIF** |
| --- | --- | --- |
| **Sustainable Project Management (SPM)** | |  |
| Economic | Econ01: Company's financial and economic performance (e.g. return on investments, solvency, and profitability) are an important factor in SPM. | 1.888 |
|  | Econ02: Company's financial benefits from good social and environmental practices (health and safety, job creation, education, training, and gains from recycling). | 1.384 |
|  |  |  |
|  | Econ03: Company's cost management (raw materials, labor, products, waste, quality, research and development, transport, and production). | 1.398 |
| Environmental | Env01: Natural resources (reducing the use of natural resources and the generation of waste, recycling, reduction of impacts and contamination of soil, biodiversity) | 1.487 |
|  | Env02: Water (consumption and use, quality, liquid waste, risks, recycling, biodiversity) | 2.103 |
|  | Env03: Energy (generation, use, distribution and transmission, global warming) | 2.306 |
| Social | Soc01: Company's labor practices management (health and safety, working conditions, diversity and opportunity, payment, benefits, and career). | 1.666 |
|  | Soc02: Company's relationships with the local community (local impacts and relations with local organizations) are forced and compulsory. | 2.103 |
|  | Soc03: Company's management of human rights (strategy and management, disciplinary procedures, collective negotiation, child labor). | 2.596 |
| **Sustainable Project Planning (SPP)** | |  |
| Managerial Control | MC01: Our project plan includes managerial control measures for project implementation. | 2.000 |
|  | MC02: We implement project tasks in accordance with the managerial control measures setting in project plan. | 2.598 |
|  | MC03: Project planning deliverables were used by a project manager to control team members sustainably. | 2.353 |
| Risk Response | RR01: Project potential risks were identified during project planning process | 1.959 |
|  | RR 02: Our project planning deliverables contain the evaluation results for potential risks. | 2.366 |
|  | RR03: Solutions for potential risks will be exported after project planning process. | 2.105 |
| Work Consensus | WC01: Project team will jointly decompose project activities during the project planning process. | 2.103 |
|  | WC02: Our project members always negotiate with the conflicting issues of project plan together. | 2.467 |
|  | WC03: Our team members acknowledge the project's baseline plan unanimously. | 2.295 |
|  | WC04: We will follow the steps from the predetermined project plan to implement the project sustainably. | 2.613 |
| **Sustainable Project Success (SPS)** | |  |
| Project Efficiency | PE01: Project's meeting the cost (Budget) | 1.872 |
|  | PE02: Projects completed on time (Within Project Schedule) | 2.750 |
|  | PE03: Project’s meeting the scope (Objectives) | 2.170 |
| Impact on Stakeholder- External | ISE01: Meeting product function and technical specifications | 2.980 |
|  | ISE02: Satisfying the customer's needs and solving the customer's problems | 2.789 |
|  | ISE03: Use of the product by the customer and improvement of the customer's quality of life | 2.154 |
| Impact on Team | IMT01: Impact on the professional lives of the team members | 1.831 |
|  | IMT02: Improvement of learning and growth | 2.717 |
|  | IMT03: Greater satisfaction and productivity of the team | 2.617 |
| Business Success | BS01: Increase in sales and market share | 2.231 |
|  | BS02: Increase in profitability | 2.133 |
|  | BS03: Return on investment, competitiveness, and market performance | 2.514 |
| Preparation for the Future | PPF01: Creation of new markets | 2.681 |
|  | PPF02: Creation of new products | 2.676 |
|  | PPF03: Creation of new technologies | 2.068 |
| Sustainability | SUS01: Realization and perpetuation of the economic benefits of the project | 1.859 |
|  | SUS02: Realization and perpetuation of the environmental benefits | 2.300 |
|  | SUS03: Realization and perpetuation of the social benefits of the project | 2.913 |
